# Proteomic analysis for the effects of non-saponin fraction with rich polysaccharide from Korean Red Ginseng on Alzheimer's disease in a mouse model

**DOI:** 10.1016/j.jgr.2022.09.008

**Published:** 2022-10-05

**Authors:** Sujin Kim, Yunkwon Nam, Min-jeong Kim, Seung-hyun Kwon, Junhyeok Jeon, Soo Jung Shin, Soyoon Park, Sungjae Chang, Hyun Uk Kim, Yong Yook Lee, Hak Su Kim, Minho Moon

**Affiliations:** a Department of Biochemistry, College of Medicine, Konyang University, Daejeon, Republic of Korea; b Research Institute for Dementia Science, Konyang University, Daejeon, Republic of Korea; c Veterans Medical Research Institute, Veterans Health Service Medical Center, Seoul, Republic of Korea; d Department of Chemical and Biomolecular Engineering (BK21 four), Korea Advanced Institute of Science and Technology (KAIST), Daejeon, Republic of Korea; e Department of Microbiology and Molecular Genetics, College of Biological Sciences, University of California, California, United States; f The Korean Ginseng Research Institute, Korea Ginseng Corporation, Daejeon, Republic of Korea

**Keywords:** Alzheimer's disease, Non-saponin fraction, Korean Red Ginseng, Proteomics, Genome-scale metabolic model

## Abstract

**Background:**

The most common type of dementia, Alzheimer's disease (AD), is marked by the formation of extracellular amyloid beta (Aβ) plaques. The impairments of axons and synapses appear in the process of Aβ plaques formation, and this damage could cause neurodegeneration. We previously reported that non-saponin fraction with rich polysaccharide (NFP) from Korean Red Ginseng (KRG) showed neuroprotective effects in AD. However, precise molecular mechanism of the therapeutic effects of NFP from KRG in AD still remains elusive.

**Methods:**

To investigate the therapeutic mechanisms of NFP from KRG on AD, we conducted proteomic analysis for frontal cortex from vehicle-treated wild-type, vehicle-treated 5XFAD mice, and NFP-treated 5XFAD mice by using nano-LC-ESI-MS/MS. Metabolic network analysis was additionally performed as the effects of NFP appeared to be associated with metabolism according to the proteome analysis.

**Results:**

Starting from 5,470 proteins, 2,636 proteins were selected for hierarchical clustering analysis, and finally 111 proteins were further selected for protein-protein interaction network analysis. A series of these analyses revealed that proteins associated with synapse and mitochondria might be linked to the therapeutic mechanism of NFP. Subsequent metabolic network analysis via genome-scale metabolic models that represent the three mouse groups showed that there were significant changes in metabolic fluxes of mitochondrial carnitine shuttle pathway and mitochondrial beta-oxidation of polyunsaturated fatty acids.

**Conclusion:**

Our results suggested that the therapeutic effects of NFP on AD were associated with synaptic- and mitochondrial-related pathways, and they provided targets for further rigorous studies on precise understanding of the molecular mechanism of NFP.

## Graphical abstract

**Figure:**
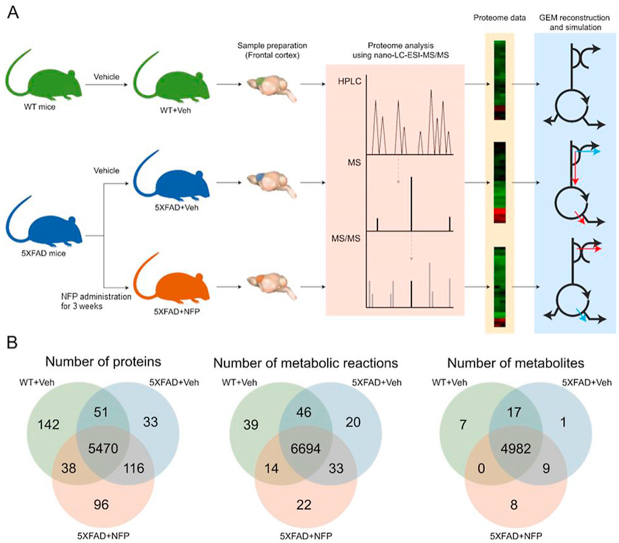

## 1 Introduction

Alzheimer's disease (AD) is the most prevailing type of dementia with complex pathologies. The extracellular accumulation of amyloid-beta (Aβ) as neuritic plaques and intracellular aggregation of hyperphosphorylated tau as neurofibrillary tangles are the major neuropathological criteria in AD [1]. The aggregation of Aβ peptides and hyperphosphorylated tau protein sequentially leads to synaptic loss and neuronal cell death [2,3]. In addition, imbalance of neurotransmitters, such as acetylcholine, norepinephrine, and dopamine in AD, is closely related to cognitive impairment and psychiatric symptoms [[4], [5], [6]]. In particular, impaired synapse in AD is characterized by decreased mitochondria and altered neurotransmitters [7]. Mitochondrial dysfunction is one of the important pathologies of AD. Structural and functional dysfunction of mitochondria appear in AD pathogenesis [8]. Therefore, strategies to mitigate Aβ-induced mitochondrial dysfunction could provide valuable insights into the treatment of AD.

A root of *Panax ginseng Meyer* contains a variety of biological activity components. The latest proteome study has demonstrated that Rg1, one of the biological activity components, changed the 49 proteins in the Aβ-treated SH-SY5Y cells [9]. As a result of protein interaction network analysis for the altered 49 proteins, the changes in protein expression levels were closely related to ribosomal proteins, mitochondria, actin cytoskeleton, and splicing proteins. Moreover, a study involving UPLC-Q/TOF-MS-based metabolomics for 3xTg mice serum administered with Rg1 showed that concentrations of 14 metabolites were changed, including inoleic acid, arachidonic acid, sphingosine, and pyruvate [10]. Importantly, ginseng polysaccharides have recently been reported as potential therapeutic agents for various nervous system diseases [11]. We reported the alleviating effects of non-saponin fraction with rich polysaccharide (NFP) from Korea red ginseng (KRG) on AD-related pathologies in the 5XFAD mouse model of AD through histological and behavioral analysis [12]. Despite the ameliorative and protective effects of NFP on cognitive decline and AD-related pathologies, such as Aβ accumulation, neuroinflammation, neuronal loss, mitochondrial dysfunction, and impaired adult hippocampal neurogenesis, mechanisms behind the therapeutic effects still remain to be further characterized.

**Fig. 1:**
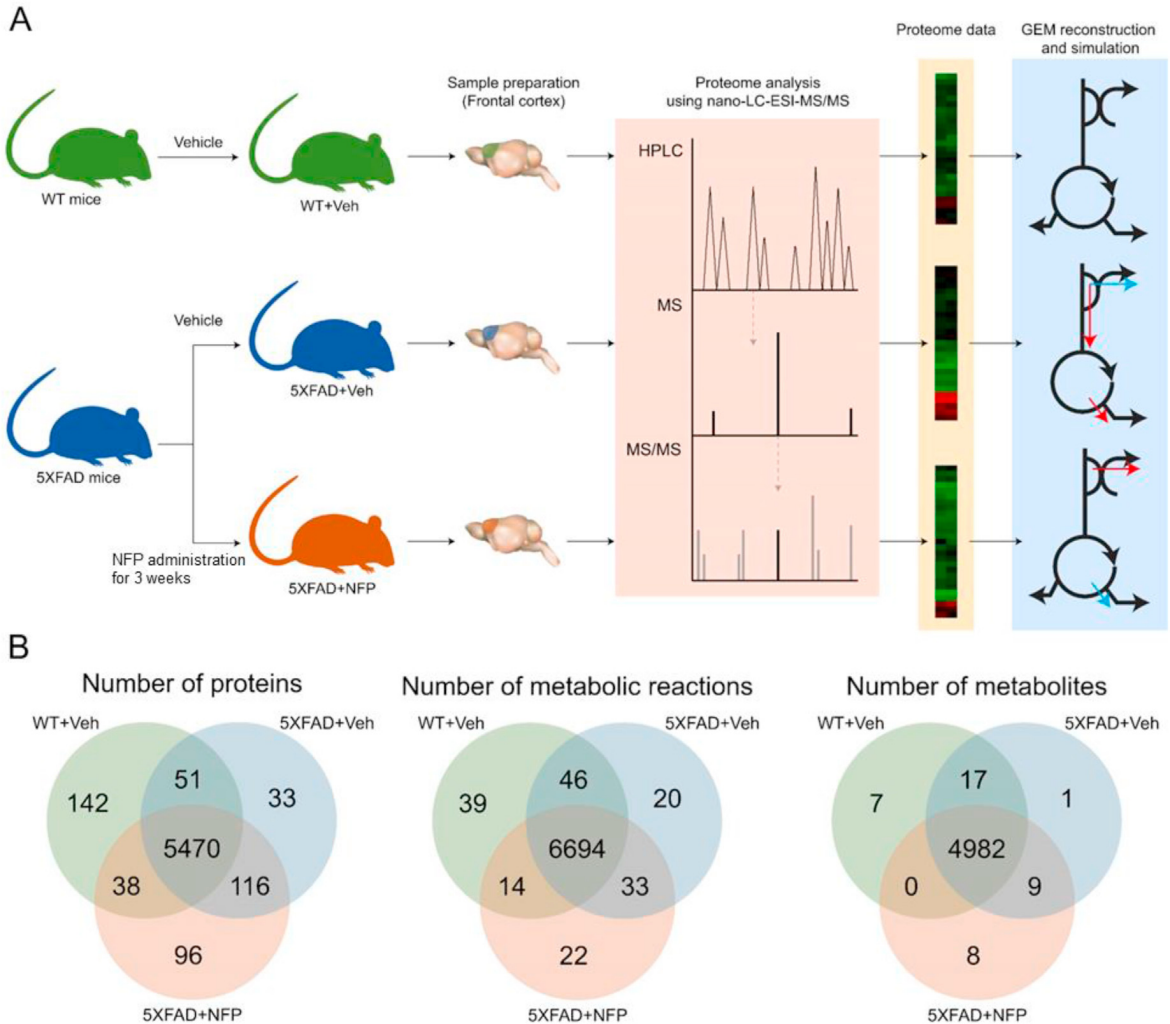
Schematic workflow of proteome and metabolic network analyses for the effects of NFP on AD. (A) Use of the vehicle-treated WT mice (WT + Veh), the vehicle-treated 5XFAD mice (5XFAD + Veh), and the NFP-treated 5XFAD mice (5XFAD + NFP) for the proteome and metabolic network analyses. Proteome and metabolic network analyses were conducted by using nano-LC-ESI-MS/MS, and genome-scale metabolic models (GEMs), respectively. (B) Venn diagrams, showing the distribution of proteins, metabolic reactions and metabolites among the three mouse groups. The same metabolites in different organelles were counted as different metabolites from the mouse group-specific GEMs.

**Fig. 2:**
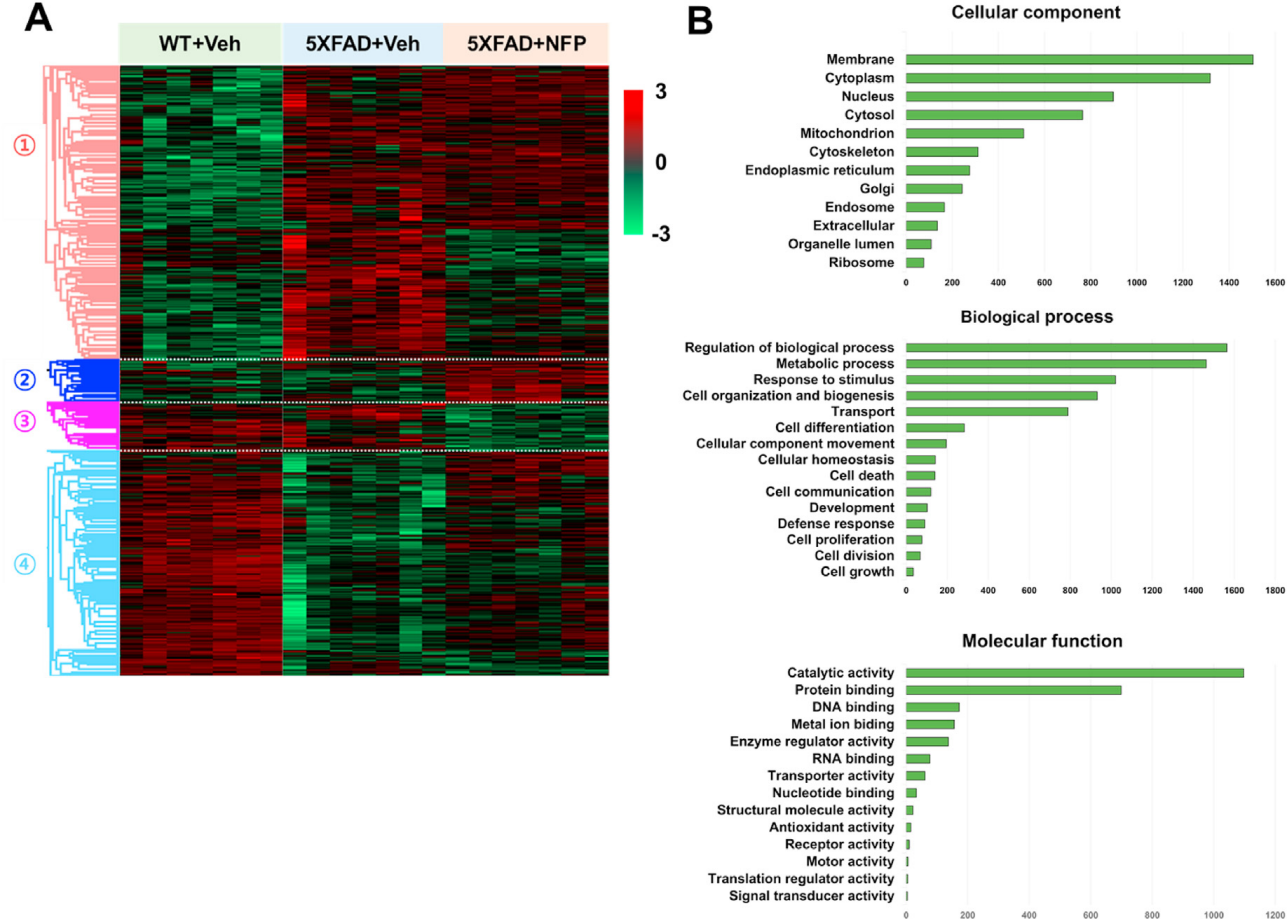
Analysis of proteome data obtained from the vehicle-treated WT mice (WT + Veh), the vehicle-treated 5XFAD mice (5XFAD + Veh), and the NFP-treated 5XFAD mice (5XFAD + NFP). (A) Hierarchical clustering of differentially expressed proteins (DEPs) among the three mouse groups (ANOVA, Benjamini-Hochberg method-based FDR < 0.05). Protein expression level is given from Z-score normalization. The DEPs were grouped into four clusters. (B) Gene Ontology (GO) analysis for 2,636 proteins with significantly altered expression levels across the three mouse groups.

A proteomic analysis reveals the functional role of a protein, the interaction between proteins, and relative protein expression levels [13]. Our previous proteomic analysis study identified the effects of NFP on alteration of protein expression levels in the aged brain [12]. Examination of the differentially expressed proteins in the aged brains treated with NFP showed that the functions of upregulated proteins were related to cellular growth and neuroprotection, while downregulated protein functions were associated with cellular death and neurodegeneration. Moreover, in the brain with AD, we demonstrated the therapeutic efficacy of NFP from KRG for the AD treatment in 5XFAD mice [12]. To grasp cluses on mechanisms underlying the therapeutic effects of NFP on AD pathogenesis, in this study, we conducted proteome analysis for the frontal cortex of 5XFAD mice after administration of NFP (Fig. 1). The proteome analysis involved the use of nano-LC-ESI-MS/MS, followed by hierarchical clustering and protein-protein interaction (PPI) network analysis. Furthermore, metabolic network analysis was additionally performed on the basis of the proteome data obtained in this study. So-called a genome-scale metabolic models (GEMs) were developed, which described each mouse group's metabolism, in order to analyze the effects of NFP at a metabolic level (Fig. 1). Here, GEM is a computational model that contains information on entire metabolic genes and reactions, and can be simulated under various environmental and genetic conditions [14]. Taken together, the integrated proteomic and metabolic network analyses provided potential targets, in particular pathways associated with mitochondria, that will serve as a basis for further rigorous studies (Fig. 2).

## 2 Materials and methods

### 2.1 Preparation of Korean Red Ginseng extract and reagents

KRG extracts (15 brix) from 6-year-old roots of *Panax ginseng Meyer* were purchased from the Korea Ginseng Corporation. Three carbohydrate-digesting enzymes, pectinesterase from orange peel, amyloglucosidase from *Aspergillus niger*, and α-amylase from *Aspergillus orizae*, were purchased from Sigma-Aldrich. Dithiothreitol (DTT), ammonium bicarbonate (NH_4_HCO_3_), formic acid (FA), urea, ammonium formate, iodoacetamide (IAA), and trifluoroacetic acid (TFA) were also obtained from Sigma-Aldrich. Polygalacturonase from *Aspergillus aculeatus* was acquired from Megazyme. Water and acetonitrile (ACN) with a high-performance liquid chromatography (HPLC) grade were obtained from JT Baker (Phillipsburg, NJ, USA), and lyophilized trypsin was purchased from Promega (Madison, WI, USA). In accordance with a previous study [15], NFP was extracted from the KRG concentrate using ethanol precipitation, enzymatic hydrolysis, and size-exclusion chromatography. The analysis of free sugars was carried out using a modified version of the method published by Joo et al [16]. The extraction of 0.1 g NFP with 10 mL of distilled water produced six free sugars: galactose, fructose, glucose, maltose, lactose, and sucrose. The NFP has 139.6 ± 2.8 mg/g galacturonic acid, 74.7 ± 4.6 mg/g galactose, and 63.4 ± 4.1 mg/g arabinose, according to the acidic polysaccharide components analysis.

### 2.2 Animal and administration

5XFAD mice (Tg6799 Stock #006554; Jackson Laboratory) have five familial AD mutations within the *PSEN1* and *APP* human transgenes. PSEN1 contains M146 and L286 mutations, while the *APP* transgene contains the Florida (I716V), Swedish (K607N and M671L), and London (V717I) mutations. These mice rapidly exhibit AD-related pathogenesis, such as Aβ deposition, neurodegeneration, and cognitive dysfunction. Genotyping was performed by polymerase chain reaction analysis of tail DNA. NFP was dissolved in saline and administered intragastrically daily for 3 weeks at a dose of 100 mg/kg. 5XFAD and wild-type (WT) mice at 3.5-month-old were used in this study. Three groups of male mice were used in the study: vehicle-treated WT mice (n = 3); vehicle-treated 5XFAD mice (n = 9); and NFP-treated 5XFAD mice (n = 6). The proper care, maintenance, and use of animals in this study followed protocols approved by the Institutional Animal Care and Use Committee of Konyang University. This animal experiment was approved by the ethics committee of Konyang University (Project identification code: P-20-16-E−01, date: 27 April 2020).

### 2.3 Protein extraction

Avertin (Tribromoethanol; Sigma-Aldrich, 250 mg/kg i.p.) was used to anesthetize the animals. Each sample (i.e., brains removed from mice) was dissolved in 100 μL of 5% SDS, reduced with 20 mM dithiothreitol in 50 mM NH_4_HCO_3_ for 10 min at 95 °C, and alkylated with 40 mM iodoacetamide in 50 mM NH_4_HCO_3_ for 30 min under the light blocking. S-TRAP™ (Protifi) was used for the fast and reproducible preparation of proteomics samples. Denatured, non-digested proteins were bound to the S-TRAP™. Each sample was incubated overnight at 37 °C with 12.5 μg sequencing grade modified trypsin/LysC (Promega) in 50 mM NH_4_HCO_3_ buffer (pH 7.8) on S-TRAP column. Eluted peptide sample was dried down and quantified. The samples were re-suspended in 0.1% formic acid and dried for LC-MS analysis.

### 2.4 Nano-LC-ESI-MS/MS analysis

The peptide samples were analyzed by UltiMate 3000 RSLCnano system that was coupled to a Q Exactive™ Plus Hybrid Quadrupole-Orbitrap™ mass spectrometer with a nano-ESI source (Thermo Fisher Scientific). Tryptic peptides from the bead column were reconstituted in 100 μL of 0.1% FA in water, and separated on an Acclaim™ Pepmap 100 C18 column (500 mm × 75 μm i.d., 3 μm, 100 Å) equipped with a C18 Pepmap trap column (20 mm × 100 μm i.d., 5 μm, 100 Å; Thermo Fisher Scientific) over 200 min (250 nL/min) using a 5–40% ACN gradient in 0.1% formic acid and 5% DMSO for 150 min (250 nL/min) at 50 °C. Mass spectra were acquired in a data-dependent mode with an automatic switch between a full scan and top 20 data-dependent MS/MS scans. The target value for the full scan MS spectra, selected from a 350 to 1800 *m*/*z*, was 3,000,000 AGS target with a maximum injection time of 100 ms and a resolution of 70,000.

### 2.5 Data search, statistical analysis, and bioinformatic analysis

**Fig. 3:**
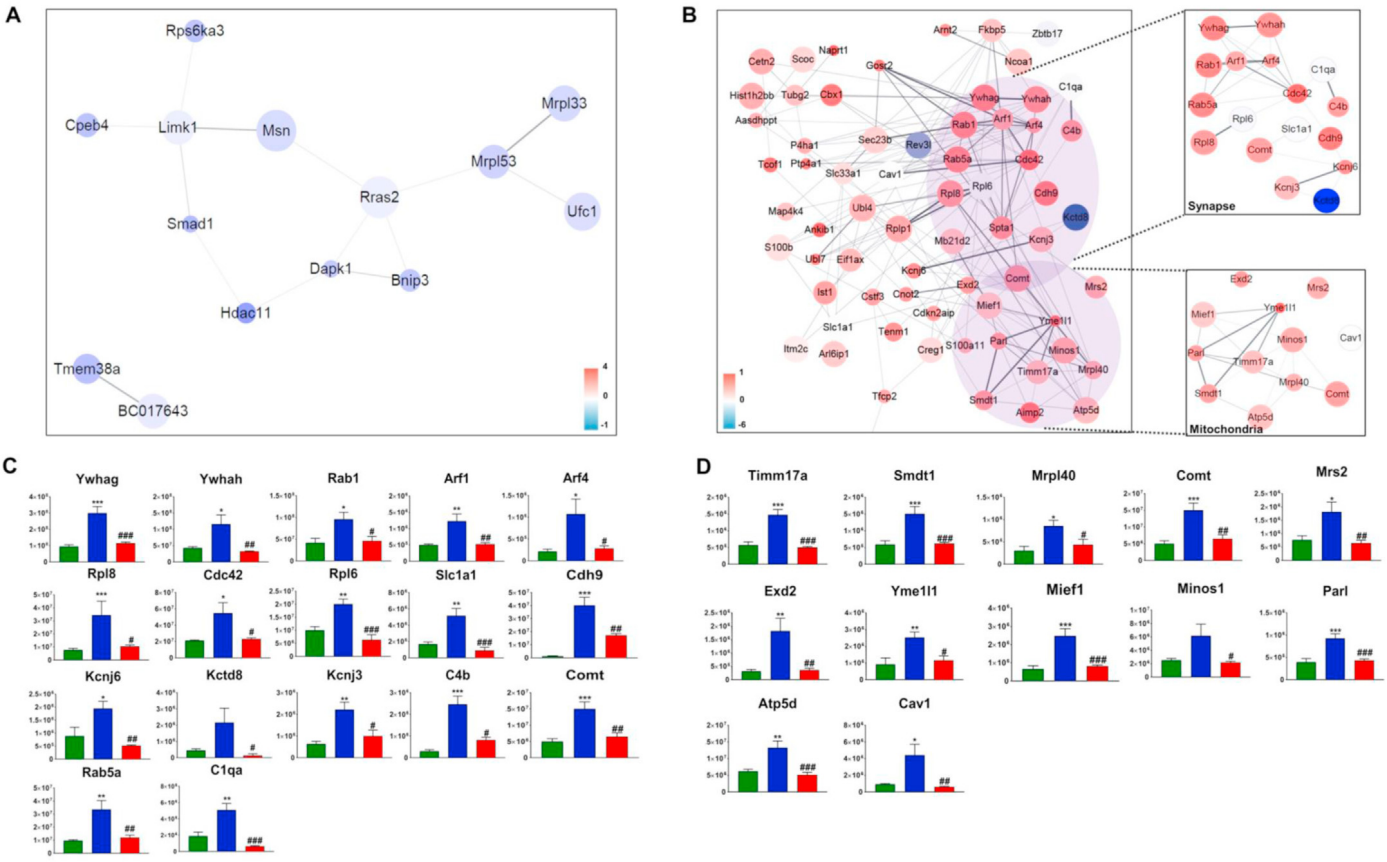
Protein-protein interaction (PPI) network analysis of the 111 proteins that were significantly regulated in the NFP-treated 5XFAD group in comparison with the vehicle-treated 5XFAD group. (A) A PPI of 14 proteins upregulated in the NFP-treated 5XFAD mice in comparison with the vehicle-treated 5XFAD mice. (B) A PPI of 70 proteins downregulated in the NFP-treated 5XFAD mice in comparison with the vehicle-treated 5XFAD mice. (C) Bar graphs showing the abundance (y-axis) of the synapse-related proteins from the vehicle-treated WT mice (green bars), the vehicle-treated 5XFAD mice (blue bars) and the NFP-treated 5XFAD mice (red bars). (D) Bar graphs showing the abundance (y-axis) of the mitochondria-related proteins from the vehicle-treated WT mice (green bars), the vehicle-treated 5XFAD mice (blue bars) and the NFP-treated 5XFAD mice (red bars). (C, D) Protein abundances are presented as the mean ± S.E.M. Statistical significance of differences between the vehicle-treated WT mice (green bars) and the vehicle-treated 5XFAD mice (blue bars) was analyzed using one-way ANOVA (*∗p* < 0.05, *∗∗p* < 0.01, and *∗∗∗p* < 0.001). Likewise, statistical significance of differences between the vehicle-treated WT mice (green bars) and the NFP-treated 5XFAD mice (red bars) was analyzed using one-way ANOVA (^*#*^*p* < 0.05, ^*##*^*p* < 0.01, and ^*###*^*p* < 0.001).

The obtained MS/MS spectra were assigned with proteins by using SequestHT on Proteome Discoverer (Version 2.4, Thermo Fisher Scientific) and the UniProt mouse database (Oct 2019) [17]. The identified proteins were analyzed and visualized (Fig. 2A) using Perseus (Version 1.6.13) [18,19]. For this, one-way analysis of variance (ANOVA), with Benjamini-Hochberg method-based false discovery rate (FDR) and the significance level of 0.05, was used to identify the significant differences in the protein expression levels among the vehicle-treated WT mice, the vehicle-treated 5XFAD mice, and the NFP-treated 5XFAD mice. Gene Ontology (GO) annotation of proteins identified from the proteome analysis was carried out by using Proteome Discoverer (Version 2.4, Thermo Fisher Scientific) (Fig. 2B). PPI network was constructed by using StringApp 1.7.1, a Cytoscape 3.8 app [20] (Fig. 3A and B). Statistical analysis for the expression levels of selected individual proteins was also performed using the GraphPad Prism 7.0 software (Fig. 3C and D). In this case, one-way ANOVA with followed by Tukey's test was conducted for comparison of the three mouse groups. *p*-value of < 0.05 was considered statistically significant for the one-way ANOVA and Tukey's post hoc tests.

### 2.6 Reconstruction, analysis and simulation of mouse group-specific genome-scale metabolic models

Mouse group-specific genome-scale metabolic models (GEMs) were developed in MATLAB R2021a (9.10.0.1739362). To generate a mouse group-specific GEM, tINIT algorithm [21] was implemented through a GitHub repository (https://github.com/SysBioChalmers/Human-GEM). Mouse1 1.2.0 was used as a generic GEM [22]. Upon generation of the mouse group-specific GEMs, a minimal set of reactions from Mouse1 1.2.0 were added to the resulting mouse group-specific GEMs to ensure the cell growth *in silico* under Ham's medium; the mouse group-specific GEMs did not show the cell growth without this correction procedure. Evaluation of the resulting mouse group-specific GEMs using MEMOTE [23] showed that they showed high qualities on the basis of 100% stoichiometric consistency, 99.4% reactions mass-balanced, 100% reactions charge-balanced, and 100% of metabolite connectivity, on average.

**Fig. 4:**
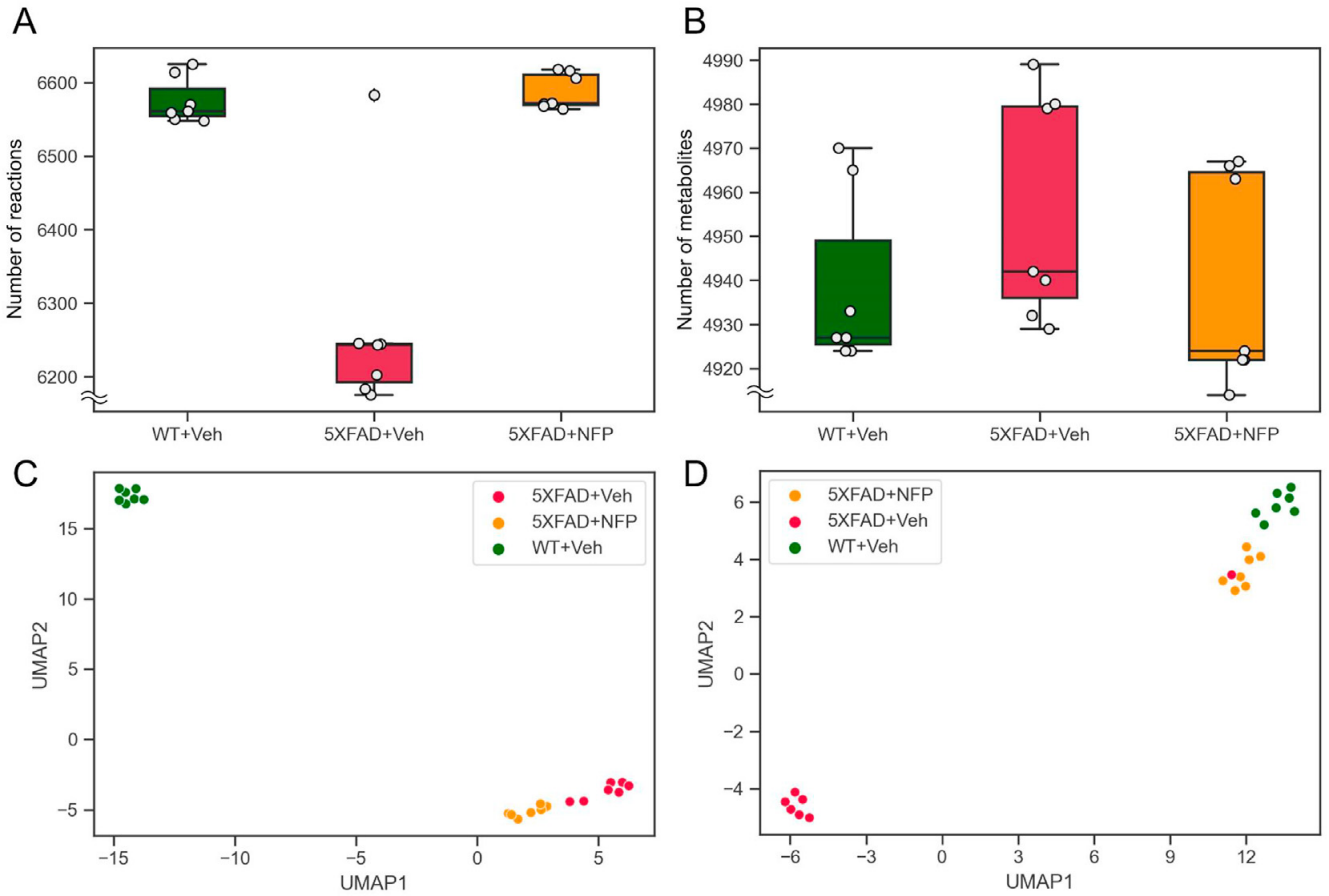
Statistics and UMAP analysis of the mouse group-specific GEMs. (A) The number of reactions in the GEMs for the vehicle-treated WT mice (WT + Veh), the vehicle-treated 5XFAD mice (5XFAD + Veh), and the NFP-treated 5XFAD mice (5XFAD + NFP). (B) The number of metabolites in the GEMs for WT + Veh, 5XFAD + Veh, and 5XFAD + NFP. The same metabolites in different organelles were counted as different metabolites. (C) UMAP projection of the proteome data from WT + Veh, 5XFAD + Veh, and 5XFAD + NFP. (D) UMAP projection of the GEMs for WT + Veh, 5XFAD + Veh, and 5XFAD + NFP.

**Fig. 5:**
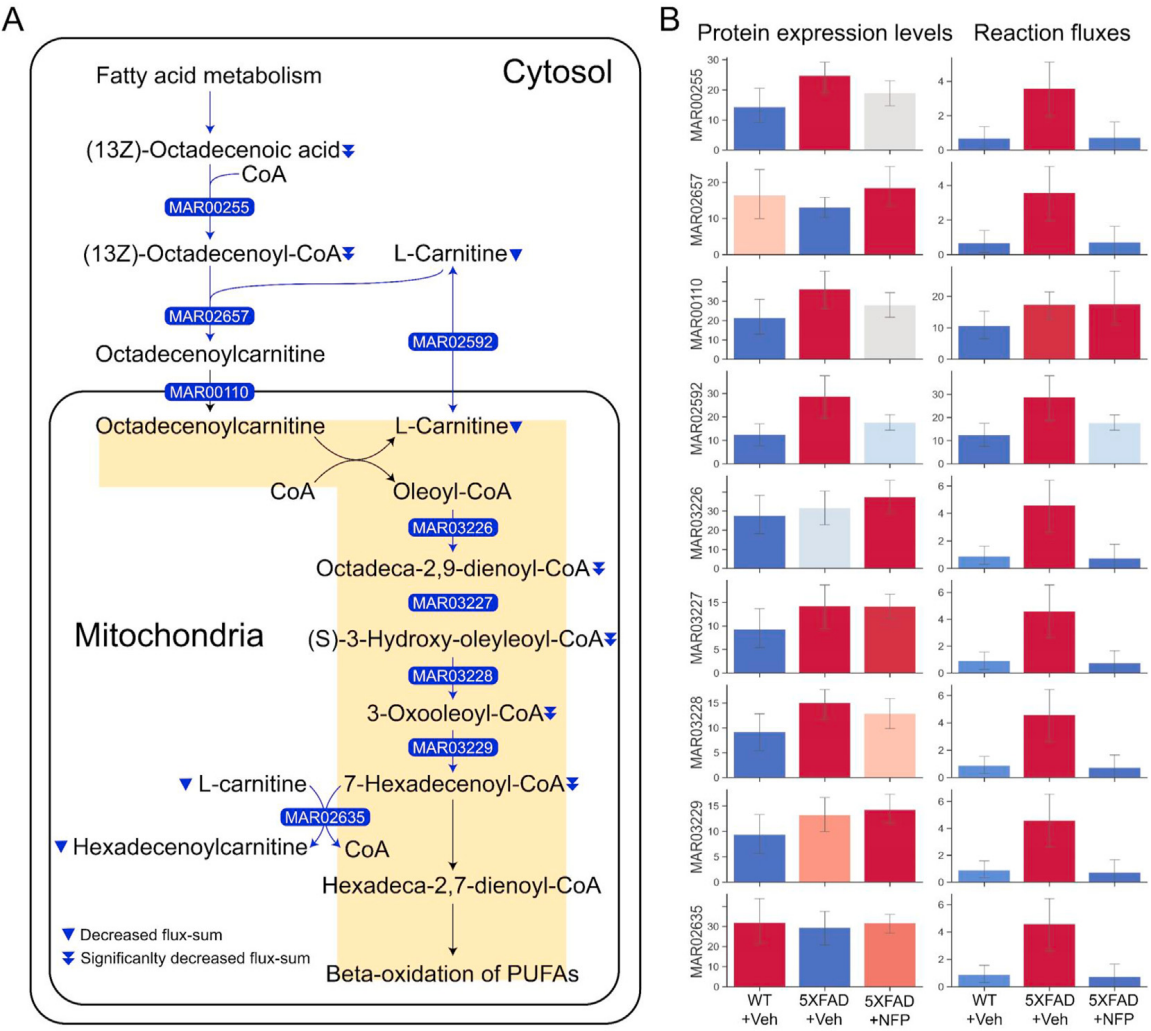
Simulation results of the mouse group-specific GEMs. (A) Mitochondrial carnitine shuttle pathway and mitochondrial beta-oxidation of polyunsaturated fatty acids (PUFAs) that showed the greatest decrease in the flux values in the NFP-treated 5XFAD mice (5XFAD + NFP) in comparison with the vehicle-treated 5XFAD mice (5XFAD + Veh). ‘Decreased flux-sum’ for a metabolite indicates that the flux-sum value was increased in the GEM of 5XFAD + Veh, compared with the vehicle-treated WT mice (WT + Veh), but decreased again in 5XFAD + NFP. If these flux changes were statistically significant according to Wilcoxon rank-sum test with a significance level of *p* < 0.05, such flux-sum values were referred to as ‘Significantly decreased flux-sum’. (B) Protein expression levels and reaction flux values near the metabolites with ‘Decreased flux-sum’ or ‘Significantly decreased flux-sum’ in (A). Error bars represent the maximum and minimum values of protein expression levels (left graphs) or reaction flux values (right graphs). Genes and pathways for the presented reactions are: MAR00255, fatty acid activation in cytosol (*Acsbg1* or *Acsbg2* or *Acsl1* or *Acsl3* or *Acsl4* or *Acls5* or *Acsl6* or *Meikin* or *Slc27a2*); MAR02657, carnitine shuttle in cytosol (*Cpt1a* or *Cpt1b* or *Cpt1c*); MAR00110, fatty acid oxidation (*Slc25a20*); MAR02592, carnitine shuttle in mitochondria (*Slc25a20* or *Slc25a29*); MAR03226, beta-oxidation of unsaturated fatty acids (n-9) in mitochondria (*Acad9* or *Acadl* or *Acadm* or *Acadvl*); MAR03227, beta-oxidation of unsaturated fatty acids (n-9) in mitochondria (*Echs1* and *Ehhadh* and *Hadha*); MAR03228, beta-oxidation of unsaturated fatty acids (n-9) in mitochondria (*Hadh* and *Hadha* and *Hsd17b10*); MAR03229, beta oxidation of unsaturated fatty acids (n-9) in mitochondria (*Acaa2* and *Hadha* and *Hadhb*); and MAR02635, carnitine shuttle in mitochondria (*Cpt2* or *Crat*).

Reading and editing of the Systems Biology Markup Language (SBML) files were implemented by using COBRApy 0.21.0 [24]. All the simulations of the mouse group-specific GEMs, including least absolute deviation (LAD) [25,26] and flux-sum prediction [27], were conducted by using in-house Python script and Gurobi Optimizer 9.1.2 (Gurobi Optimization). Mouse group-specific GEMs were analyzed using UMAP 0.5.1 [28], and GEM-related features were presented using seaborn 0.11.0 [29] (Fig. 4). For hyperparameters of UMAP, ‘number of neighbors’ was set to be 5 (Fig. 4C) and 11 (Fig. 4D) as a result of examining the values between 3 and 15. Other hyperparameters were set to their default values. To compare the three mouse groups with respect to reaction fluxes and metabolite flux-sum values (Fig. 5), Wilcoxon rank-sum test was implemented by using SciPy 1.4.1 [30].

## 3 Results and discussion

### 3.1 Proteome profiles as a result of Alzheimer's disease and NFP treatment

To understand the effects of NFP on protein alterations in a brain with AD, we performed a proteomic analysis in the brains of 5XFAD mice treated with NFP, and age-matched controls (i.e., 5XFAD and WT mice) treated with vehicle. Protein samples were extracted from the vehicle-treated WT mice, the vehicle-treated 5XFAD mice, and the NFP-treated 5XFAD mice, and were subjected to nano-LC-ESI-MS/MS analysis (Fig. 1A). In the frontal cortex, our proteomic evaluations identified a total of 5,701 proteins from the vehicle-treated WT mice, 5,670 proteins from the vehicle-treated 5XFAD mice, and 5,720 proteins from the NFP-treated 5XFAD mice; 5,470 proteins were commonly identified in all the three groups (probability for a protein to be assigned with MS peaks > 99%; and probability for a peptide to be assigned with MS peaks > 95 %) (Fig. 1B). Among all the identified proteins, these 5,470 proteins were considered for further analysis as they were identified reproducibly from the three groups. In particular, within the 5,470 proteins, 2,636 proteins in the frontal cortex were further selected, which showed the Benjamini-Hochberg method-based FDR of < 0.05. These 2,636 proteins were subjected to subsequent hierarchical clustering and PPI network analyses. As further elaborated below, GEMs of the three mouse groups appeared to have different number of reactions and metabolites in accordance with protein distributions (Fig. 1B)

### 3.2 Hierarchical clustering analysis of the proteome data from the three mouse groups

Hierarchical clustering analysis was conducted for the 2,636 proteins in the frontal cortex, which generated four clusters as a result (Fig. 2A). We divided the analyzed proteome into four clusters (Table S1). Cluster 1 is a protein affected by AD, which is increased proteins in 5XFAD compared to WT. Cluster 2 and 3 are proteins that are not affected by AD. Proteins belonging to Cluster 2 were decreased in both WT and 5XFAD, and proteins belonging to Cluster 3 were increased in both WT and 5XFAD. Finally, Cluster 4 is a protein affected by AD, which is a decreased protein in 5XFAD compared to WT. In particular, expression levels of some proteins in Clusters 1 and 4 in the vehicle-treated 5XFAD mice became more similar to the WT group as a result of the NFP treatment. Thus, the proteins belonging to Clusters 1 and 4 may serve as therapeutic targets of NFP. Indeed, several proteins that were reported to be affected by NFP showed consistent changes in their expression levels in our proteome data. For example, clathrin heavy chain 1 (Cltc) and Ras-related protein Rab-1B (Rab1b), both from Cluster 1, were downregulated by NFP [15], and also dystonin (Dst), myosin-10 (Myh10), and Rho GTPase-activating protein 35 (Arhgap35), all belonging to Cluster 4, were reported to be upregulated in response to NFP [15].

GO terms associated with the 2,636 proteins in the 5XFAD group showed that these proteins are mainly associated with membrane (23.3 %) and cytoplasm (20.4 %) for the cellular component domain, regulation of biological process (22.2 %) and metabolic process (20.8 %) for the biological process domain, and catalytic activity (43.9 %) and protein binding (28 %) for the molecular function domain (Fig. 2B and Fig. S1-S4). In particular, high association with metabolic process and catalytic activity for the NFP-regulated proteins suggests that the proteome data also need to be analyzed in the context of metabolism as presented below. Meanwhile, proteins belonging to Clusters 2 and 3 appeared to be not affected by AD, but interestingly, affected by the NFP treatment, which resulted in greater deviations in their expression levels from the vehicle-treated WT mice; these unexpected changes in the protein expression levels may suggest some possible side-effects incurred by the NFP (Fig. 2A).

To further narrow down the positive effects of NFP on AD, subsequent PPI network analysis was conducted with focus on protein clusters in the NFP-treated 5XFAD mice, expression levels of which became more similar to the vehicle-treated WT mice. For this, one-way ANOVA was conducted among the vehicle-treated WT, the vehicle-treated 5XFAD mice, and NFP-treated 5XFAD mice in order to find specific proteins that showed statistically significant changes in their expression levels as a result of NFP treatment. Consequently, 111 proteins could be identified from the 2,636 proteins, which appeared to be significantly affected by NFP (Tables S2 and S3). These 111 proteins included 28 proteins whose expression levels were upregulated in the NFP-treated 5XFAD mice compared with the vehicle-treated 5XFAD mice (Table S2), and 83 proteins with downregulated expression levels for the two same groups (Table S3). These 111 proteins were subjected to the PPI network analysis.

### 3.3 PPI network analysis of the three mouse groups

To gain further insights into these 111 proteins, the upregulated proteins in the NFP-treated 5XFAD mice in comparison with the vehicle-treated 5XFAD mice, and the downregulated proteins for the same two groups were separately considered to construct PPI networks using StringApp [20] (Fig. 3). A PPI network involving 14 upregulated proteins, and another PPI network with 70 downregulated proteins were subsequently constructed; the remaining 27 proteins failed to be connected to either PPI network. The PPI network with all the 14 upregrulated proteins revealed a strong relation with cellular component (Fig. 3A and Table S2), but did not show notable clusters. In contrast, the PPI network with the downregulated proteins showed two clear clusters, which were associated with synapse and mitochondria (Fig. 3B and Table S3). In our previous study, proteins associated with synapse and mitochondria were also found to be associated with the therapeutic effects of NFP [12]. Indeed, expression levels of the proteins associated with synapse (Fig. 3C) and those with mitochondria (Fig. 3D) appeared to be downregulated in the NFP-treated 5XFAD mice. Representative synapse-related proteins associated with AD involve the YWHA family, such as tyrosine 3-monooxygenase (Ywhag) and Ywhah, which play an important role in neuronal and synaptic development, function, and plasticity [31,32]; the YWHA family proteins were detected together with neurofibrillary tangles in the brain of AD [33]. Moreover, a previous study showed that the YWHA family protein increased GSK3β-dependent phosphorylation of tau [34]. Moreover, 14-3-3 proteins, such as Ywhag and Ywhah, are key proteins involved in regulation of transmission and plasticity, apoptosis, response to stress, and memory and learning. In post-mortem studies of AD patients, it has been reported that these proteins are elevated in various brain regions [35]. For a mitochondria-related protein, YME1L1 is an ATPase from the AAA family that contributes to the preservation of mitochondrial morphology [36]. A previous study reported that the YME1L1 level was increased in the frontal cortex of familial AD patients than in sporadic AD patients [37]. Particularly, the increased YME1L1 level could induce cell death and mitochondrial dysfunction [36,38]. Meanwhile, catechol-O-methyltransferase (Comt) protein was the only one found in both two clusters on synase and mitochondria, which was abnormally increased in the vehicle-treated 5XFAD; this protein was downregulated in the NFP-treated 5XFAD mice. Comt is an enzyme involved in the degradation of catecholamines in the synapse of the cerebral cortex, and it plays a role in cognitive function [39].

### 3.4 Reconstruction and analysis of the mouse group-specific GEMs

Because the NFP-regulated proteins in the 5XFAD group appeared to be associated with metabolic process and catalytic activity according to the GO and PPI network analyses, potential metabolic effects of NFP on AD were further examined by using mouse group-specific GEMs. The mouse group-specific GEMs describe metabolism (i.e., metabolic gene-protein-reaction associations) of the vehicle-treated WT mice, the vehicle-treated 5XFAD mice, and the NFP-treated 5XFAD mice, and they were reconstructed by using the corresponding proteome data as well as Mouse1, a generic GEM of *Mus musculus* [22] (Materials and methods). Interestingly, statistics of the mouse group-specific GEMs showed that the GEMs presenting the NFP-treated 5XFAD mice showed greater similarities with the vehicle-treated WT mice than the vehicle-treated 5XFAD mice, in terms of the number of reactions and metabolites (Fig. 4A and B); the GEMs for the vehicle-treated WT mice, the vehicle-treated 5XFAD mice, and the NFP-treated 5XFAD mice had an average of 6,588 reactions and 4,940 metabolites, 6,268 reactions and 4,956 metabolites, and 6,575 reactions and 4,939 metabolites, respectively. This observation indicates that NFP may be heavily affecting the structure of a metabolic network of the 5XFAD mice.

Further dimensionality reduction analysis using UMAP showed some intriguing patterns regarding the effects of NFP on AD at proteome (Fig. 4C) and metabolic levels (Fig. 4 D). At the proteome level, a cluster presenting the NFP-treated 5XFAD mice was more closely located to a cluster for the vehicle-treated 5XFAD mice than the vehicle-treated WT mice (Fig. 4C). This unexpected clustering outcome might be partly attributed to the highly expressed proteins in Cluster 1 from the hierarchical clustering of the proteome data (red pixels in Cluster 1 of Fig. 2A). In contrast to the proteome level, the GEMs for the vehicle-treated WT mice and the NFP-treated 5XFAD mice were clustered more together, while a cluster for the vehicle-treated 5XFAD mice was away from these two clusters. This analysis suggests that the mouse group-specific GEMs can provide additional evidences for the effects of NFP in addition to proteome, and that NFP may exert its therapeutic effects on metabolic pathways in the 5XFAD mice by restoring the expression levels of proteins to the levels found in the normal mice.

### 3.5 Simulation of the mouse group-specific GEMs

To gain better insights into metabolic responses of the NFP-treated 5XFAD mice in comparison with the vehicle-treated 5XFAD mice, their corresponding mouse group-specific GEMs were simulated. For this, least absolute deviation (LAD) was performed to predict intracellular metabolic flux values in each mouse group [25]. As a result, flux changes were observed in several metabolic pathways in response to the NFP treatment where the NFP-treated 5XFAD mice appeared to have flux values more similar to the vehicle-treated WT mice than the vehicle-treated 5XFAD mice. Representative metabolic pathways predicted to be affected by NFP included transport reactions, retinol metabolism, purine metabolism, mitochondrial beta-oxidation of polyunsaturated fatty acids (PUFAs), mitochondrial carnitine shuttle pathway, and cholesterol metabolism. For the transport reactions, those associated with the transport of amino acids showed significant flux changes, for example solute carrier family 7 (cationic amino acid transporter, Slc7a6), solute carrier organic anion transporter family member 1A4 (Slco1a4) and Solute carrier family 13 member 5 (Slc13a5); Slco1a4 and Slc13a5 belong to Cluster 1 from the hierarchical clustering of the proteome data (Fig. 2A).

The most notable flux changes were found in the mitochondrial carnitine shuttle pathway and mitochondrial beta-oxidation of PUFAs (Fig. 5A). These two pathways showed overall reaction fluxes significantly increased in the vehicle-treated 5XFAD mice, compared to the vehicle-treated WT mice, and decreased in the NFP-treated 5XFAD mice. These flux changes in response to the NFP treatment were overall consistent with the proteome profiles, but appeared to be more dramatic (Fig. 5B). Among a total of 27 genes involved in the cytosolic and mitochondrial fatty acid pathways (Fig. 5A), 23 genes were found to be covered by the proteome data (Fig. 2A). For these 23 genes, seven genes were located in Cluster 1 from the hierarchical clustering of the proteome data (Fig. 2A), and six genes (*Acsl5*, *Slc27a2*, *Slc25a20*, *Hadha*, *Hadh*, and *Hsd17b10*) out of these seven genes appeared to be associated with protein expression levels (Fig. 2A) and metabolic fluxes (Fig. 5A) that showed the same changing patterns in response to the NFP treatment; protein expression levels or metabolic flux values for these six genes were decreased in the NFP-treated 5XFAD mice in comparison with the vehicle-treated 5XFAD mice. These six genes encode the following proteins: *Acsl5*, long-chain-fatty-acid--CoA ligase 5; *Slc27a2*, very long-chain acyl-CoA synthetase; *Slc25a20*, mitochondrial carnitine/acylcarnitine carrier protein; *Hadha*, trifunctional enzyme subunit alpha, mitochondrial; *Hadh*, hydroxyacyl-coenzyme A dehydrogenase, mitochondrial; and *Hsd17b10*, 3-hydroxyacyl-CoA dehydrogenase type-2. The metabolic simulation results presented herein were consistent with previous studies on mitochondrial metabolic disorder and fatty acid oxidation that have been reported to be linked with the AD pathogenesis [40,41].

On the basis of the metabolic flux values predicted, so-called flux-sum values were also calculated to identify biologically important metabolites in these mitochondrial carnitine shuttle pathway and mitochondrial beta-oxidation of PUFAs in the context of AD; here, flux-sum is defined to be the sum of all the fluxes necessary for the generation of a metabolite, and indicates the overall importance of that metabolite in a cell [27]. Subsequently, metabolites with significant flux-sum changes as a result of the NFP treatment were identified, including (13Z)-octadecenoic acid, (13Z)-octadecenoyl-CoA and L-carnitine from cytosol, and octadeca-2,9-dienoyl-CoA, (S)-3-hydroxy-oleyleoyl-CoA, 3-oxooleoyl-CoA and 7-hexadecenoyl-CoA from mitochondria (Fig. 5A). These metabolites may serve as potential biomarkers when examining the effects of NFP and other relevant treatment strategies.

In this study, we revealed that NFP treatment exerts therapeutic effects by modulating synaptic- and mitochondrial-associated proteins in the AD brain. Moreover, we propose a study to investigate the molecular mechanism of NFP on pathogenic factors other than Aβ in brains with AD-like pathology. Nevertheless, our findings show that the potential molecular mechanism underlying the NFP therapeutic effect of KRG on AD-related alterations in synaptic and mitochondrial-associated protein pathways.

## Declaration of competing interest

The authors declare that they have no known competing financial interests or personal relationships that could have appeared to influence the work reported in this paper.
