# Supplementary material for: Proteomic analysis for the effects of non-saponin fraction with rich polysaccharide from Korean Red Ginseng on Alzheimer's disease in a mouse model
Source: J Ginseng Res. 2022 Oct 5;47(2):302–10. doi: 10.1016/j.jgr.2022.09.008 (PMC10014184; doi:10.1016/j.jgr.2022.09.008)
Supplement: Multimedia component 1 [file mmc1.docx]

**Supplementary figures**

**
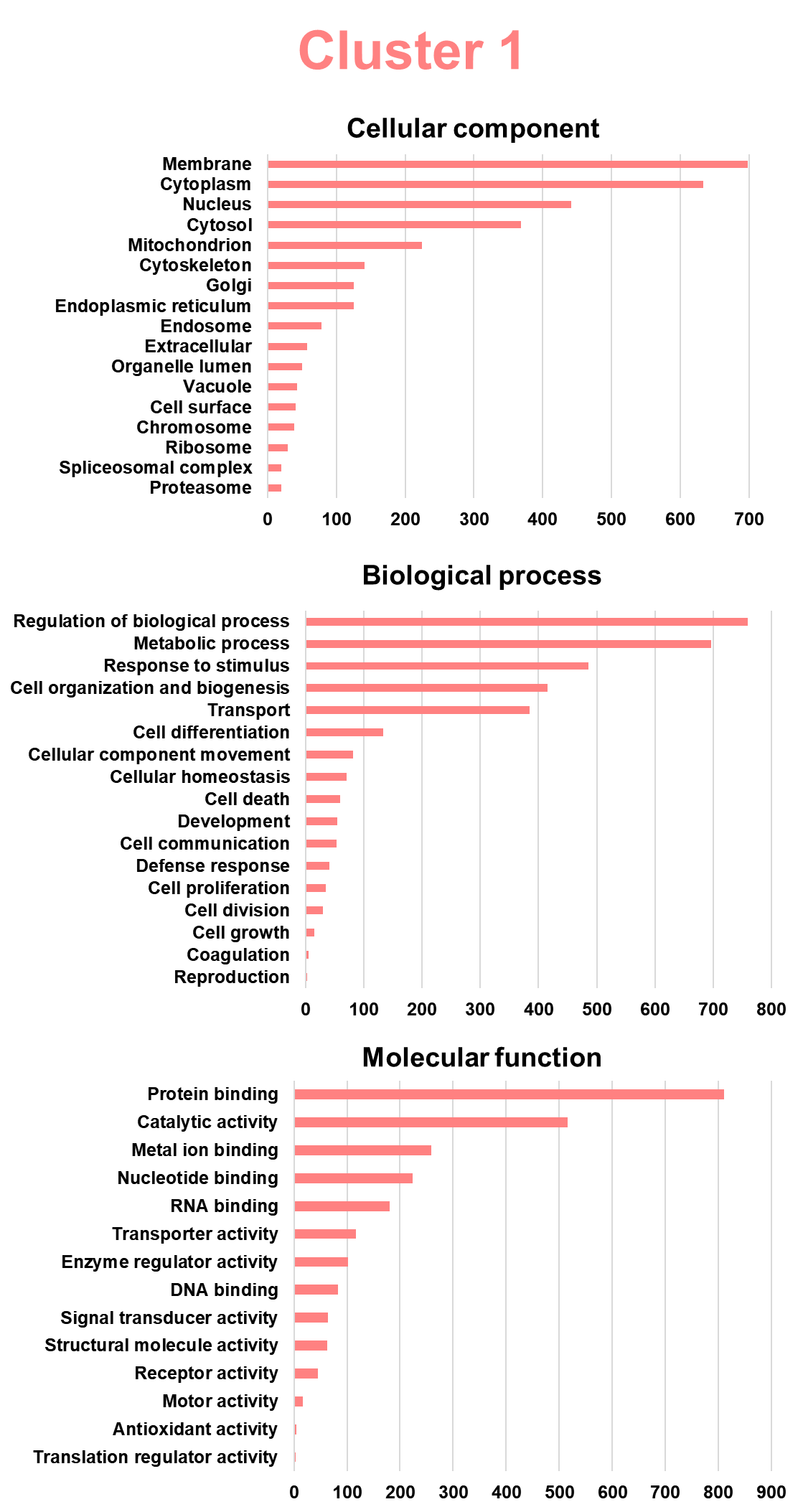
**

**Supplementary figure 1.** Gene Ontology (GO) analysis of 1,273 proteins belonging to Cluster 1. These proteins are mainly associated with membrane (22.3 %) and cytoplasm (20.2 %) for the cellular component domain, regulation of biological process (22.8 %) and metabolic process (20.9 %) for the biological process domain, and protein binding (32.6 %) and catalytic activity (20.7 %) for the molecular function domain.


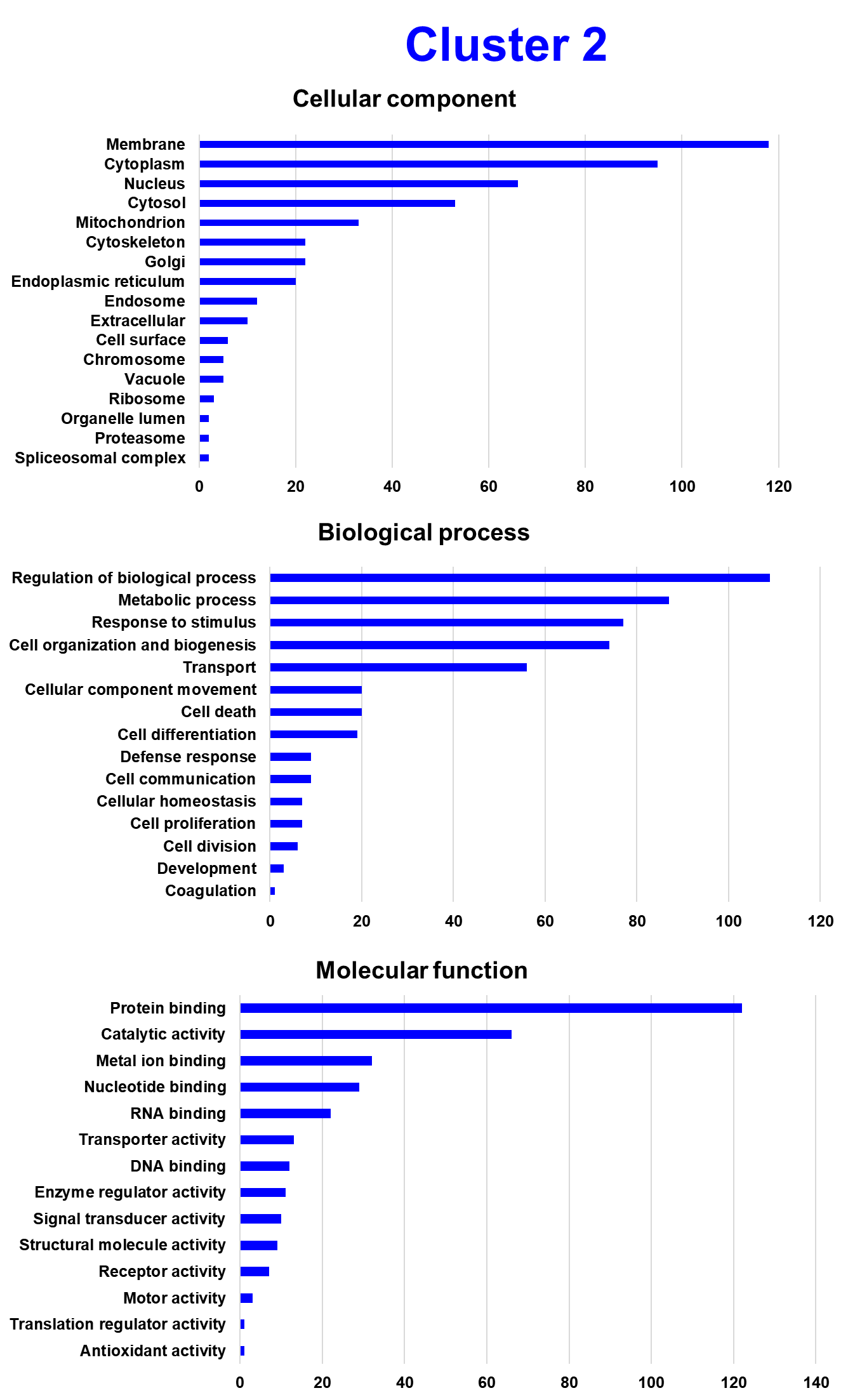


**Supplementary figure 2.** Gene Ontology (GO) analysis of 184 proteins belonging to Cluster 2. These proteins are mainly associated with membrane (24.8 %) and cytoplasm (20.0 %) for the cellular component domain, regulation of biological process (21.6 %) and metabolic process (17.2 %) for the biological process domain, and protein binding (36.1 %) and catalytic activity (19.5 %) for the molecular function domain.


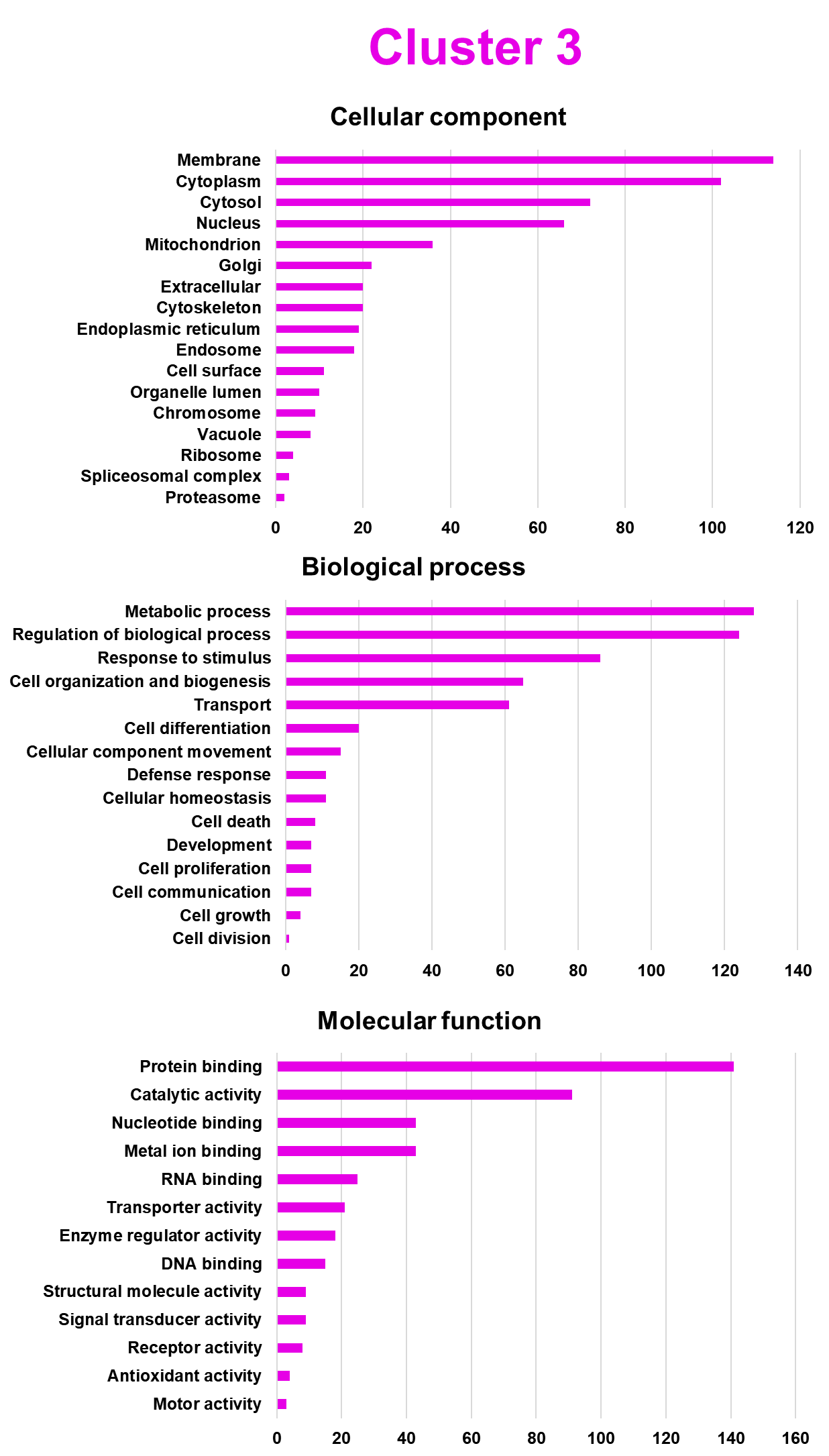


**Supplementary figure 3.** Gene Ontology (GO) analysis of 206 proteins belonging to Cluster 3. These proteins are mainly associated with membrane (21.3 %) and cytoplasm (19.0 %) for the cellular component domain, metabolic process (23.0 %) and regulation of biological process (22.3 %) for the biological process domain, and protein binding (32.8 %) and catalytic activity (21.1 %) for the molecular function domain.


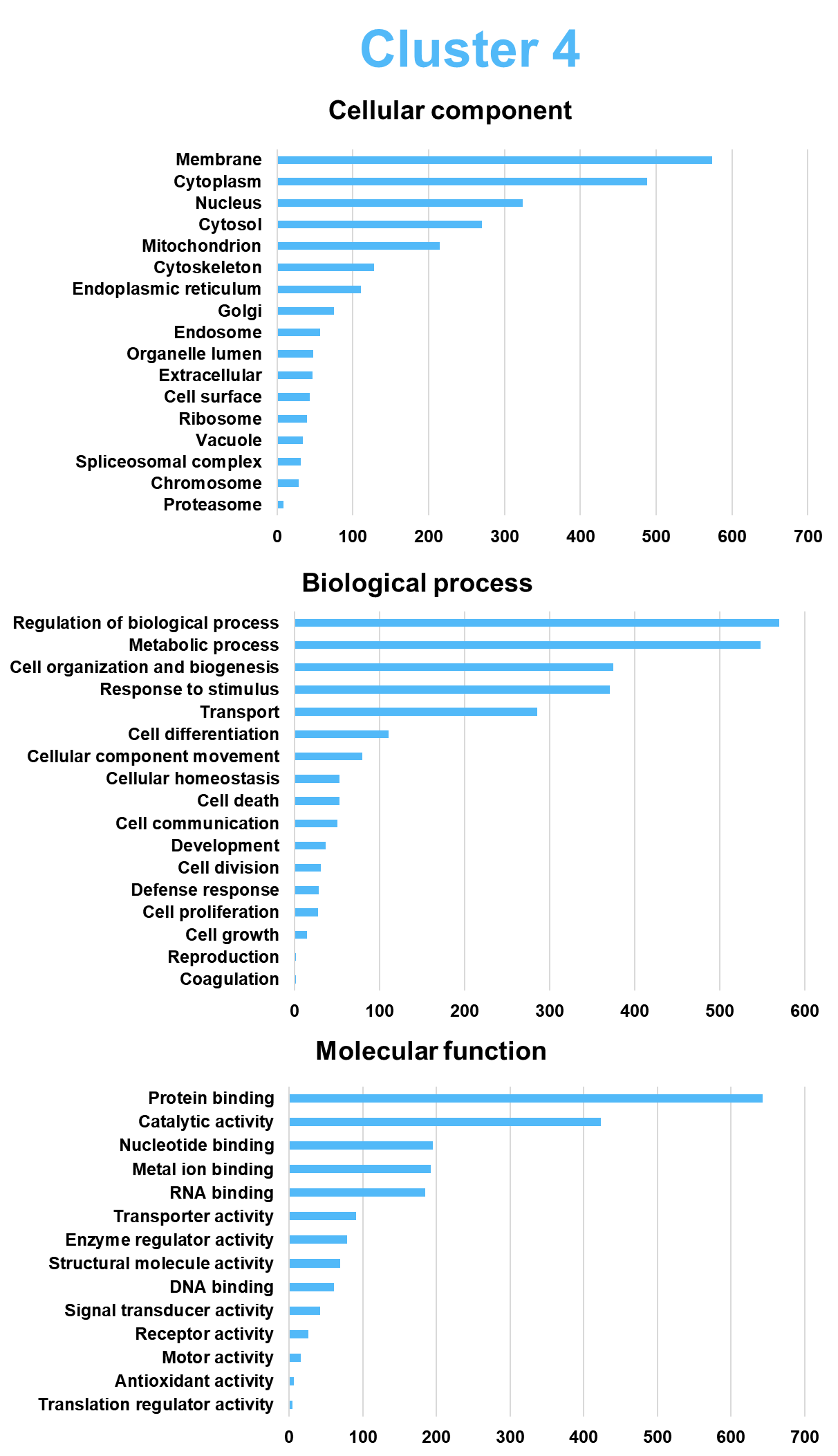


**Supplementary figure 4.** Gene Ontology (GO) analysis of 970 proteins belonging to Cluster 4. These proteins are mainly associated with membrane (22.7 %) and cytoplasm (19.3 %) for the cellular component domain, regulation of biological process (21.6 %) and metabolic process (20.7 %) for the biological process domain, and protein binding (31.6 %) and catalytic activity (21.0 %) for the molecular function domain.
