# Supplementary material for: Proteomic analysis for the effects of non-saponin fraction with rich polysaccharide from Korean Red Ginseng on Alzheimer's disease in a mouse model
Source: J Ginseng Res. 2022 Oct 5;47(2):302–10. doi: 10.1016/j.jgr.2022.09.008 (PMC10014184; doi:10.1016/j.jgr.2022.09.008)
Supplement: Multimedia component 2 [file mmc2.docx]

**Supplementary table 1.** The clustering criterion

| **Cluster 1** | **[Proteins altered by AD]**  Increased protein in AD brain  Increased proteins in 5XFAD compared to WT |
| --- | --- |
| **Cluster 2** | **[Proteins not affected by AD]**  Decreased proteins in both WT and 5XFAD |
| **Cluster 3** | **[Proteins not affected by AD]**  Increased proteins in both WT and 5XFAD |
| **Cluster 4** | **[Proteins altered by AD]**  Decreased protein in AD brain  Decreased proteins in 5XFAD compared to WT |

**Supplementary table 2.** List of proteins in which protein expression reduced by AD pathologies was reversed by NFP treatment

| **Accession number** | **Description** | **Gene** | **Biofunction** | ***p*-value** |
| --- | --- | --- | --- | --- |
| C0HK80 | Adipocyte-related X-chromosome expressed sequence 2 | Arxes2 | Cell differentiation  Metabolic process  Transport | 0.05 |
| O55003 | BCL2/adenovirus E1B 19 kDa protein-interacting protein 3 | Bnip3 | Cell death  Cell organization and biogenesis  Defense response  Metabolic process  Regulation of biological process  Response to stimulus | 0.05 |
| P18654 | Ribosomal protein S6 kinase alpha-3 | Rps6ka3 | Cell death  Metabolic process  Regulation of biological process  Response to stimulus | 0.01 |
| P26041 | Moesin | Msn | Cell differentiation  Cell organization and biogenesis  Cell proliferation  Cellular component movement  Regulation of biological process  Response to stimulus | 0.01 |
| P53668 | LIM domain kinase 1 | Limk1 | Metabolic process  Regulation of biological process  Response to stimulus | 0.01 |
| P59326 | YTH domain-containing family protein 1 | Ythdf1 | Regulation of biological process | 0.001 |
| P62071 | Ras-related protein R-Ras2 | Rras2 | Cell differentiation  Regulation of biological process  Response to stimulus | 0.001 |
| P63239 | Neuroendocrine convertase 1 | Pcsk1 | Metabolic process  Regulation of biological process  Response to stimulus | 0.01 |
| P70340 | Mothers against decapentaplegic homolog 1 | Smad1 | Cell organization and biogenesis  Cell proliferation  Defense response  Metabolic process  Regulation of biological process  Response to stimulus | 0.05 |
| Q3THF9 | Coenzyme Q-binding protein COQ10 homolog B, mitochondrial | Coq10b | Metabolic process | 0.001 |
| Q3TMP8 | Trimeric intracellular cation channel type A | Tmem38a | Transport | 0.001 |
| Q3TYS2 | Cytochrome b-245 chaperone 1 | Cybc1 |  | 0.01 |
| Q7TN98 | Cytoplasmic polyadenylation element-binding protein 4 | Cpeb4 | Cell communication  Metabolic process  Regulation of biological process  Response to stimulus | 0.01 |
| Q7TNC4 | Putative RNA-binding protein Luc7-like 2 | Luc7l2 | Cell organization and biogenesis | 0.001 |
| Q80YE7 | Death-associated protein kinase 1 | Dapk1 | Cell death  Metabolic process  Regulation of biological process  Response to stimulus | 0.05 |
| Q810A3 | Tetratricopeptide repeat protein 9C | Ttc9c |  | 0.01 |
| Q8BG30 | Negative elongation factor A | Nelfa | Metabolic process  Regulation of biological process | 0.05 |
| Q8BJU2 | Tetraspanin-9 | Tspan9 | Regulation of biological process  Response to stimulus | 0.01 |
| Q8VE38 | Oxidoreductase NAD-binding domain-containing protein 1 | Oxnad1 | Metabolic process | 0.01 |
| Q91W96 | Anaphase-promoting complex subunit 4 | Anapc4 | Cell division  Metabolic process  Regulation of biological process | 0.01 |
| Q91WA3 | Histone deacetylase 11 | Hdac11 | Cell organization and biogenesis  Metabolic process  Regulation of biological process | 0.001 |
| Q923S9 | Ras-related protein Rab-30 | Rab30 | Cell organization and biogenesis  Regulation of biological process  Response to stimulus | 0.001 |
| Q9CQP0 | 39S ribosomal protein L33 | Mrpl33 | Metabolic process | 0.001 |
| Q9CQU3 | Protein RER1 | Rer1 | Cell organization and biogenesis  Regulation of biological process  Transport | 0.001 |
| Q9CR09 | Ubiquitin-fold modifier-conjugating enzyme 1 | Ufc1 | Metabolic process  Response to stimulus | 0.05 |
| Q9CRA9 | FGFR1 oncogene partner 2 homolog | Fgfr1op2 | Response to stimulus | 0.05 |
| Q9D1H8 | 39S ribosomal protein L53 | Mrpl53 |  | 0.001 |

**Supplementary table 3.** List of proteins in which protein expression increased by AD pathologies was reversed by NFP treatment

| **Accession number** | **Description** | **Gene** | **Biofunction** | ***p*-value** |
| --- | --- | --- | --- | --- |
| A2AV25 | Fibrinogen C domain-containing protein 1 | Fibcd1 |  | 0.001 |
| D3YZI9 | PiggyBac transposable element-derived protein 5 | Pgbd5 | Metabolic process | 0.01 |
| O08784 | Treacle protein | Tcof1 | Cell organization and biogenesis  Metabolic process  Regulation of biological process | 0.001 |
| O35166 | Golgi SNAP receptor complex member 2 | Gosr2 | Cell organization and biogenesis  Transport | 0.01 |
| O88587 | Catechol O-methyltransferase | Comt | Cell communication  Metabolic process  Regulation of biological process  Response to stimulus | 0.01 |
| O88668 | Protein CREG1 | Creg1 | Regulation of biological process | 0.05 |
| O88967 | ATP-dependent zinc metalloprotease YME1L1 | Yme1l1 | Cell organization and biogenesis  Cell proliferation  Metabolic process  Regulation of biological process  Response to stimulus | 0.01 |
| P01029 | Complement C4-B | C4b | Defense response  Metabolic process  Regulation of biological process  Response to stimulus | 0.05 |
| P08032 | Spectrin alpha chain, erythrocytic 1 | Spta1 | Cell organization and biogenesis  Metabolic process  Regulation of biological process | 0.001 |
| P19258 | Protein Mpv17 | Mpv17 | Cell organization and biogenesis  Metabolic process  Regulation of biological process  Response to stimulus | 0.001 |
| P21126 | Ubiquitin-like protein 4A | Ubl4a | Transport | 0.001 |
| P28667 | MARCKS-related protein | Marcksl1 | Regulation of biological process | 0.05 |
| P47911 | 60S ribosomal protein L6 | Rpl6 | Cell organization and biogenesis  Metabolic process | 0.001 |
| P47955 | 60S acidic ribosomal protein P1 | Rplp1 | Metabolic process  Regulation of biological process | 0.0 |
| P48542 | G protein-activated inward rectifier potassium channel 2 | Kcnj6 | Metabolic process  Regulation of biological process  Transport | 0.01 |
| P49817 | Caveolin-1 | Cav1 | Cell differentiation  Cell organization and biogenesis  Cellular homeostasis  Metabolic process  Regulation of biological process  Response to stimulus  Transport | 0.001 |
| P50114 | Protein S100-B | S100b | Cell differentiation  Cellular homeostasis  Metabolic process  Regulation of biological process  Response to stimulus | 0.001 |
| P50543 | Protein S100-A11 | S100a11 | Regulation of biological process | 0.001 |
| P51906 | Excitatory amino acid transporter 3 | Slc1a1 | Cell organization and biogenesis  Regulation of biological process  Transport | 0.001 |
| P60755 | MAM domain-containing glycosylphosphatidylinositol anchor protein 2 | Mdga2 |  | 0.01 |
| P60766 | Cell division control protein 42 homolog | Cdc42 | Cell communication  Cell differentiation  Cell organization and biogenesis  Cellular component movement  Metabolic process  Regulation of biological process  Response to stimulus  Transport | 0.05 |
| P61458 | Pterin-4-alpha-carbinolamine dehydratase | Pcbd1 | Cell organization and biogenesis  Metabolic process  Regulation of biological process | 0.001 |
| P61750 | ADP-ribosylation factor 4 | Arf4 | Cellular component movement  Metabolic process  Regulation of biological process  Response to stimulus  Transport | 0.05 |
| P61982 | 14-3-3 protein gamma | Ywhag | Metabolic process  Regulation of biological process  Response to stimulus  Transport | 0.001 |
| P62821 | Ras-related protein Rab-1A | Rab1A | Cell organization and biogenesis  Cellular component movement  Defense response  Metabolic process  Regulation of biological process  Response to stimulus  Transport | 0.05 |
| P62918 | 60S ribosomal protein L8 | Rpl8 | Metabolic process  Response to stimulus | 0.05 |
| P63250 | G protein-activated inward rectifier potassium channel 1 | Kcnj3 | Regulation of biological process  Response to stimulus  Transport | 0.05 |
| P68510 | 14-3-3 protein eta | Ywhah | Cell organization and biogenesis  Metabolic process  Regulation of biological process  Response to stimulus  Transport | 0.01 |
| P70365 | Nuclear receptor coactivator 1 | Ncoa1 | Cell communication  Cell organization and biogenesis  Metabolic process  Regulation of biological process  Response to stimulus  Transport | 0.01 |
| P70407 | Cadherin-9 | Cdh9 | Cell organization and biogenesis | 0.01 |
| P83917 | Chromobox protein homolog 1 | Cbx1 | Regulation of biological process | 0.01 |
| P84078 | ADP-ribosylation factor 1 | Arf1 | Cell organization and biogenesis  Cellular homeostasis  Regulation of biological process  Response to stimulus  Transport | 0.01 |
| P97820 | Mitogen-activated protein kinase kinase kinase kinase 4 | Map4k4 | Cell organization and biogenesis  Metabolic process  Regulation of biological process  Response to stimulus | 0.01 |
| P98086 | Complement C1q subcomponent subunit A | C1qa | Defense response  Metabolic process  Regulation of biological process  Response to stimulus | 0.001 |
| Q3TPE9 | Ankyrin repeat and MYND domain-containing protein 2 | Ankmy2 | Regulation of biological process | 0.01 |
| Q3U308 | Cytoplasmic tRNA 2-thiolation protein 2 | Ctu2 | Metabolic process | 0.05 |
| Q3UEI1 | cAMP-specific 3',5'-cyclic phosphodiesterase 4C | Pde4c | Metabolic process  Regulation of biological process  Response to stimulus | 0.001 |
| Q3UNZ8 | Quinone oxidoreductase-like protein 2 | Cryzl2 | Metabolic process | 0.001 |
| Q50H33 | BTB/POZ domain-containing protein KCTD8 | Kctd8 | Cell organization and biogenesis  Regulation of biological process | 0.05 |
| Q5NCE8 | Magnesium transporter MRS2 homolog | Mrs2 | Metabolic process  Transport | 0.01 |
| Q5XJY4 | Presenilins-associated rhomboid-like protein | Parl | Metabolic process  Regulation of biological process | 0.001 |
| Q60715 | Prolyl 4-hydroxylase subunit alpha-1 | P4ha1 | Cell organization and biogenesis  Metabolic process | 0.001 |
| Q60821 | Zinc finger and BTB domain-containing protein 17 | Zbtb17 | Development  Metabolic process  Regulation of biological process | 0.05 |
| Q61324 | Aryl hydrocarbon receptor nuclear translocator 2 | Arnt2 | Metabolic process  Regulation of biological process  Response to stimulus | 0.05 |
| Q61493 | DNA polymerase zeta catalytic subunit | Rev3l | Metabolic process  Response to stimulus | 0.01 |
| Q63739 | Protein tyrosine phosphatase type IVA 1 | Ptp4a1 | Development  Metabolic process  Regulation of biological process | 0.01 |
| Q641K1 | Cytosolic carboxypeptidase 1 | Agtpbp1 | Cell differentiation  Cell organization and biogenesis  Metabolic process  Response to stimulus | 0.05 |
| Q64378 | Peptidyl-prolyl cis-trans isomerase FKBP5 | Fkbp5 | Metabolic process | 0.05 |
| Q64475 | Histone H2B type 1-B | H2bc3 | Cell organization and biogenesis | 0.001 |
| Q6PHZ5 | Putative RNA-binding protein 15B | Rbm15b | Metabolic process  Regulation of biological process | 0.001 |
| Q6ZPS6 | Ankyrin repeat and IBR domain-containing protein 1 | Ankib1 | Metabolic process  Regulation of biological process | 0.01 |
| Q78YZ6 | Short coiled-coil protein | Scoc | Regulation of biological process | 0.05 |
| Q7TNS2 | MICOS complex subunit Mic10 | Micos10 |  | 0.05 |
| Q8BGP5 | Transmembrane protein 127 | Tmem127 | Cell organization and biogenesis  Regulation of biological process | 0.001 |
| Q8BGV8 | Mitochondrial dynamics protein MID51 | Mief1 | Cell organization and biogenesis  Regulation of biological process  Response to stimulus | 0.001 |
| Q8BI72 | CDKN2A-interacting protein | Cdkn2aip | Regulation of biological process  Response to stimulus | 0.01 |
| Q8C525 | Protein MB21D2 | Mb21d2 |  | 0.01 |
| Q8C5L3 | CCR4-NOT transcription complex subunit 2 | Cnot2 | Cell differentiation  Development  Metabolic process  Regulation of biological process | 0.001 |
| Q8CC86 | Nicotinate phosphoribosyltransferase | Naprt | Metabolic process  Response to stimulus | 0.001 |
| Q8K124 | Pleckstrin homology domain-containing family O member 2 | Plekho2 |  | 0.001 |
| Q8R010 | Aminoacyl tRNA synthase complex-interacting multifunctional protein 2 | Aimp2 | Cell death  Cell differentiation  Cell organization and biogenesis  Development  Metabolic process  Regulation of biological process | 0.001 |
| Q8VCK3 | Tubulin gamma-2 chain | Tubg2 | Cell organization and biogenesis | 0.01 |
| Q8VEG4 | Exonuclease 3'-5' domain-containing protein 2 | Exd2 | Metabolic process  Response to stimulus | 0.01 |
| Q91VK4 | Integral membrane protein 2C | Itm2c | Cell differentiation  Regulation of biological process | 0.001 |
| Q91W67 | Ubiquitin-like protein 7 | Ubl7 | Transport | 0.001 |
| Q99J27 | Acetyl-coenzyme A transporter 1 | Slc33a1 | Regulation of biological process  Response to stimulus  Transport | 0.01 |
| Q99LI7 | Cleavage stimulation factor subunit 3 | Cstf3 | Metabolic process | 0.01 |
| Q9CQD1 | Ras-related protein Rab-5A | Rab5a | Cell organization and biogenesis  Metabolic process  Regulation of biological process  Response to stimulus  Transport | 0.01 |
| Q9CQF6 | L-aminoadipate-semialdehyde dehydrogenase-phosphopantetheinyl transferase | Aasdhppt | Metabolic process | 0.05 |
| Q9CX00 | IST1 homolog | Ist1 | Cell division  Regulation of biological process  Transport | 0.001 |
| Q9D3D9 | ATP synthase subunit delta, mitochondrial | Atp5f1d | Metabolic process  Transport | 0.001 |
| Q9D662 | Protein transport protein Sec23B | Sec23b | Transport | 0.05 |
| Q9DB10 | Essential MCU regulator, mitochondrial | Smdt1 | Cellular homeostasis  Transport | 0.001 |
| Q9ERA0 | Alpha-globin transcription factor CP2 | Tfcp2 | Metabolic process  Regulation of biological process | 0.01 |
| Q9ET22 | Dipeptidyl peptidase 2 | Dpp7 | Metabolic process | 0.001 |
| Q9JIG4 | Protein phosphatase 1 regulatory subunit 3F | Ppp1r3f | Regulation of biological process | 0.01 |
| Q9JKW0 | ADP-ribosylation factor-like protein 6-interacting protein 1 | Arl6ip1 | Cell death  Cell organization and biogenesis  Regulation of biological process  Transport | 0.001 |
| Q9R1K9 | Centrin-2 | Cetn2 | Cell division  Cell organization and biogenesis  Metabolic process  Regulation of biological process  Response to stimulus | 0.05 |
| Q9WTS4 | Teneurin-1 | Tenm1 | Cell organization and biogenesis  Metabolic process  Regulation of biological process  Response to stimulus | 0.001 |
| Q9Z0V8 | Mitochondrial import inner membrane translocase subunit Tim17-A | Timm17a | Transport | 0.001 |
| Q3TY60 | Protein FAM131B | Fam131b |  | 0.001 |
| Q8BMJ3 | Eukaryotic translation initiation factor 1A | Eif1ax | Metabolic process | 0.05 |
| Q9Z2Q5 | 39S ribosomal protein L40 | Mrpl40 |  | 0.05 |
